# Vortex-assisted magnetic solid phase extraction of Pb and Cu in some herb samples on magnetic multiwalled carbon nanotubes

**DOI:** 10.3906/kim-2009-26

**Published:** 2021-02-17

**Authors:** Dilek KAMAŞ, Aslıhan KARATEPE, Mustafa SOYLAK

**Affiliations:** 1 Department of Chemistry, Faculty of Arts and Science, Nevşehir Hacı Bektaş Veli University, Nevşehir Turkey; 2 Department of Chemistry, Faculty of Sciences, Erciyes University, Kayseri Turkey

**Keywords:** Magnetic solid phase extraction, multiwalled carbon nanotubes, preconcentration, flame atomic absorption spectrometry, copper, lead

## Abstract

This study is the development of a new solid phase extraction method based on using magnetic multiwalled carbon nanotubes impregnated with 1-(2-pyridylazo)2-naphthol (PAN) for separation, preconcentration, and flame atomic absorption spectrometric determination of Pb(II) and Cu(II). Optimization of the method was done by investigating pH effect, amount of magnetic multiwalled carbon nanotubes impregnated with PAN, eluent type and volume, matrix effects, and volume of the sample. The optimum adsorbent amount was found to be 75 mg and the optimum pH value was found as 5.5. The detection limits were 16.6 μg L^-1^ for Pb(II) and 18.9 μg L^-1^ for Cu(II). The relative standard deviations (RSD%) were less than 4%. Two certified reference materials: SPS-WW2 wastewater and NCS-DC73349 (bush branches and leaves) were used to test the validation of the method. The method was successfully applied to the analysis of Pb(II) and Cu(II) ions in daisy, mint, paprika, sage, rosemary, daphne leaves, heather, green tea, and*Viburnum opulus*samples.

## 1. Introduction

In recent years, people tend to use some herbal teas as an alternative medicine to keep their health good or to heal the diseases, such as cancer, depression, liver diseases, colds, insomnia, and diabetes [1]. Herbal medicines are popular due to their perceived effectiveness in treating and preventing the disease, and it is believed that these treatments are natural and not harmful [2]. Daisy, mint, sage, rosemary, daphne leaves, heather, and green tea are commonly used for this purpose. Herbal teas can be defined as the herbal mixture made from their leaves, seeds, and/or roots of various plants [3].

Depending on the soil where these plants are grown and the water source used for irrigation, the plants can contain undesirable and/or undeclared substances as radioactive particles, pesticide residues, polycyclic aromatic hydrocarbons (PAHs), fumigants, and microbes including pathogens, mycotoxins, and heavy metals. These contaminants can be accumulated in herbs in different stages such as cultivation, storage, and their processing may cause adverse effects on the health of the consumers [4-6]. Thus, the analyses of such plants and spices is of great importance due to the risk of intake of hazardous materials because of overconsumption. Heavy metal contamination in herbs and the determination of its level is a great concern for chemists. Metals such as Al, Co, Cr, Cu, Fe, Mn, Ni, and Zn are essential plant nutrients; however, they may become toxic at higher concentrations [7]. Some activities also increase the concentrations of heavy metals in the environment such as, mining, metal ore smelting, industrial activities, and using pesticides and fertilizers in agriculture. Plants can accumulate heavy metals from the soil where they are planted as well as from the water source used for their irrigation or surrounding air. When the plants are grown in the contaminated soil without knowing whether they were contaminated or not, toxic heavy metals can enter into the food chain of human by accumulating in the plant [8,9]. European Commission considers mercury, nickel, lead, arsenic, and cadmium as major contaminants for the environment [10].

There are various papers reporting the works for the metal analysis in the herbs and spices by using different methods. These methods are: flame or electrothermal atomic absorption spectrometry, atomic fluorescence spectrometry, voltammetry, and inductively coupled plasma optical emission spectrometry [11,12]. The determination of trace elements such as aluminum, arsenic, cadmium, antimony, lead, iron, barium, zinc, manganese, copper, nickel, chromium, mercury and vanadium in various plant leaves and flowers, their teas, tinctures, and herbal medicine was carried out by different researchers [13-19]. When the concentrations of the metal ions in the sample are lower than the detection limit of the method, using a preconcentration method before determination becomes very important. Carbon nanotubes (CNTs) are used as novel solid phase extractors for various inorganic and organic materials at low concentrations. The structure of the CNTs can be defined as graphite sheet, which is rolled into a tube. They have different types such as multiwalled (MWNTs), single-walled (SWCNTs), or double-walled (DWCNTs) carbon nanotubes due to the number of carbon atom sheets in the nanotubes’ wall. Because of their extremely high surface/volume ratio, they are suitable adsorbents for many organic and inorganic materials in the preconcentration and separation studies, and they have been used for the purpose of metal ions’ preconcentration in many works [20-24]. Due to the rapid and effective technology, magnetic materials are attractive adsorbents for many scientists for separation, and their usage in solid phase extraction is a novel subject. Using magnetic carbon nanotubes (M-CNTs) as magnetic solid phase provides sample pretreatment procedures, which are optimum and selective with some advantages such as shorter preparation time, minimized matrix effects, and less sample-preparation steps. Carbon nanotubes were used to remove, adsorb, or preconcentrate different environmental pollutants after modification with different magnetic materials. When it is compared with the other solid phase extraction methods, in magnetic solid phase extraction methods, sample pretreatment is very simple because there is no need to package the column with the adsorbent. Moreover, in case of batch mode operation, the phase can be separated quickly and easily by applying an external magnetic field [25-30].

In this work, nano-sized M-MWCNTs were prepared, and then impregnated with PAN, and used for the SPE of Pb(II) and Cu(II) in commonly consumed herbs such as daisy, mint, sage, daphne leaves,*Viburnum opulus*, rosemary, heather, green tea and paprika, which are used as tea and spice in Turkey. The determinations were made by using a flame AAS.

## 2. Experimental

### 2.1. Instrumentation

The pH values of all solutions were measured by using a digital pH meter (Sartorius PT-10). The solutions were mixed by a vortex mixer (VWR int., Germany). A Sonorex ultrasonic bath was used in the magnetic carbon nanotubes synthesis stage. The determinations of Pb(II) and Cu(II) ions were performed by flame atomic absorption spectrometer (Perkin-Elmer Inc., Waltham, MA USA, Analyst 300).

### 2.2. Chemicals and reagents

Unless specified, in all experimental studies analytical grade reagents and purified water (with a Milli-Q system, Millipore, USA) were used. M-MWCNTs and 1-(2-pyridylazo)-2-naphthol (PAN) were supplied form Sigma-Aldrich (St. Louis MO, USA). Nitric acid and acetone were purchased from Merck (Darmstadt, Germany). For the pH adjustments, phosphate buffers were used in the pH range of 4.0-7.0 and ammonium/ammonia buffer solutions were used for the pH range of 8.0-9.0.

### 2.3. Preparation of the magnetic M-MWCNTs impregnated with PAN

Nano-sized magnetic carbon nanotubes were prepared by using analytical grade FeCl_2_.4H_2_O, FeCl_3_.6H_2_O and ammonia (d: 0,903 g/cm³, 25%) solution. Coprecipitation method is used due to its easiness and high-volume capacity. This method is based on the coprecipitation of the dissolved Fe^2+^ and Fe^3+^ ions in aqua media by adding a base solution. According to this reaction, an initial concentration ratio of Fe^3+^: Fe^2+^ was 2 : 1 made by dissolving 0.3825 gram of FeCl_3_.6H_2_O and 0.7425 gram FeCl_2_.4H_2_O in a solution having a volume of 40 mL. After adding 0.25 g of MWNT to this solution, the mixture was heated on a magnetic stirrer under the argon atmosphere at 75 °C for 20 min. Then, 10 mL of NH_3_ was added as drops while the solution was being stirred continuously. This procedure took approximately an hour, then the formation of the magnetic M-MWCNTs was seen by the black precipitate. The gained magnetic property of the product was checked by using a neodymium permanent magnet. Then, it was washed by distilled water until obtaining a pH value around 7 and dried in an oven. Saturation of these magnetic M-MWCNTs with PAN was made by adding 25 mL PAN (0.2 %w/v) in ethanol and mixing for 3 h. The final product was then dried and ground before using.

### 2.4. Vortex-assisted magnetic solid phase extraction method (VA-MSPE) for preconcentration

**Figure 1:**
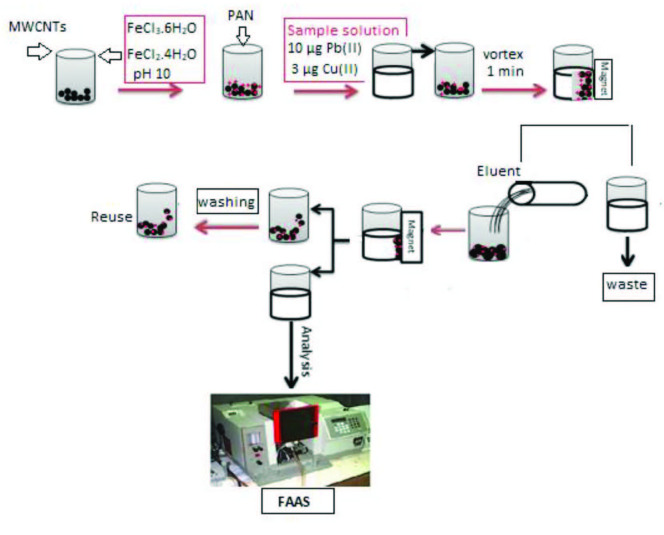
The schematic diagram of the presented vortex assisted magnetic solid phase extraction procedure.

A magnetic solid phase extraction method assisted by vortex was carried out by adding 10 µg Pb(II), 3 µg Cu(II) in a working solution (10 mL) that contains 75 mg of magnetic MCNT impregnated with PAN and 2 mL of pH 5.5 phosphate buffer solution. The solution was mixed for 2 min by a vortex, then, a strong neodymium permanent magnet was used for the separation of the adsorbent, which has gained magnetic property and the supernatants were separated by decantation. 3 mL HNO_3_ in 10% acetone solution was used for elution by adding it to the adsorbent. Then, the mixture was mixed for 1 min on vortex to desorb the analytes. After isolating the extract from the adsorbent with the neodymium magnet, the absorbances were measured by a flame AAS. The schematic diagram of the M-MWCTs solid phase extraction procedure is depicted in Figure 1.

### 2.5. Sample preparation

The developed preconcentration method was applied to the determination of Pb(II) and Cu(II) in various herb samples, and the method is also applied to certificated SPS-WW2 wastewater and NCS-DC 73349 (bush branches and leaves). A total of 1.0 g of the herb samples and standard reference material were weighed and evaporated to dryness at approximately 100 °C by adding 10 mL HNO_3_. Then, 10 mL of HNO_3_ and 5 mL of H_2_O_2_ were added to the residual, and it was again evaporated to dryness at approximately 100 °C on a hotplate. The samples were filtered through a blue band filter paper, then diluted to a volume of 25 mL with distilled water, and the developed solid phase extraction method was applied successfully to determine Pb(II) and Cu(II) in herbs and spices such as daisy, mint, sage, daphne leaves,*Viburnum opulus*, rosemary, heather, green tea, and paprika

## 3. Results

### 3.1. Effects of the pH value

**Figure 2:**
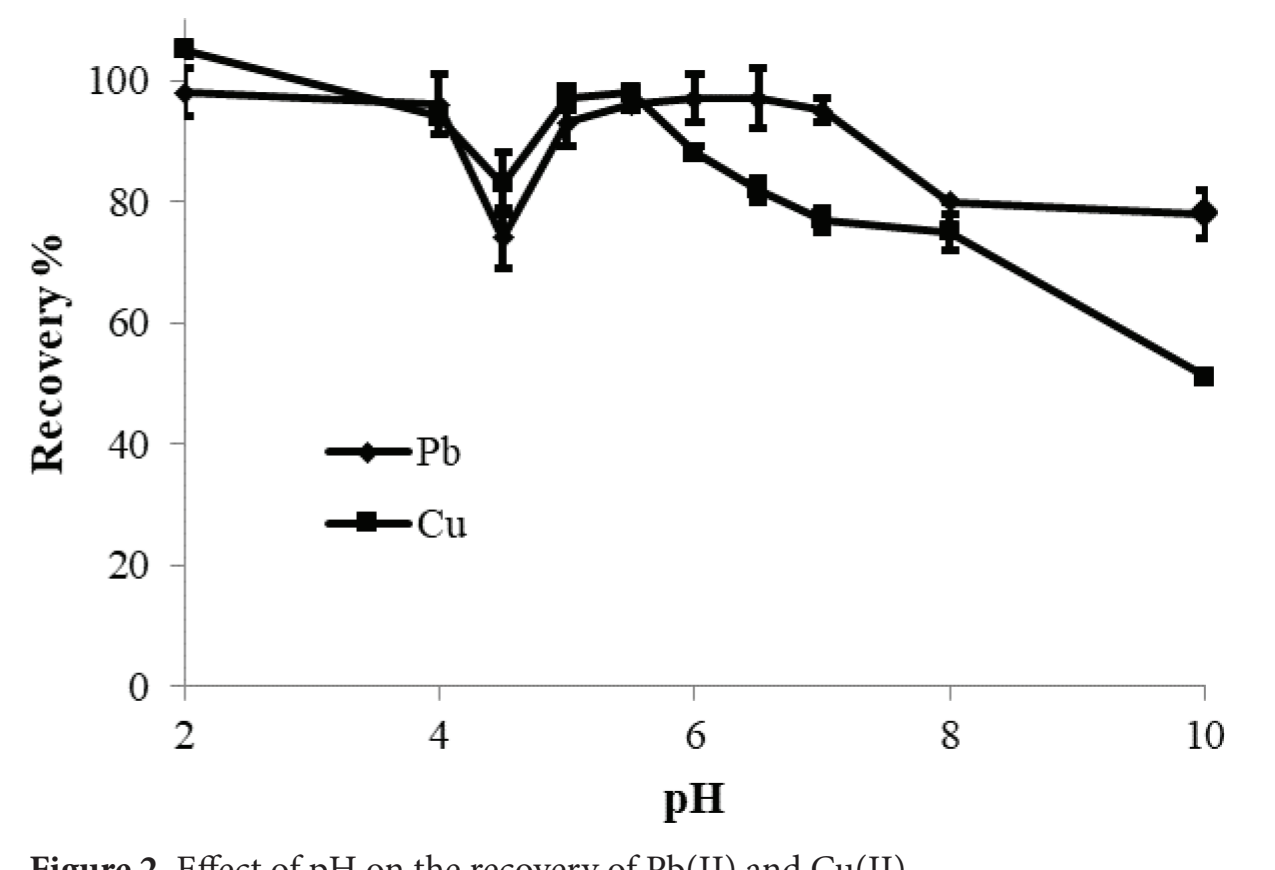
Effect of pH on the recovery of Pb(II) and Cu(II).

Since the pH value affects the complexation of the metal ions, it was the first parameter to be optimized. Thus, pH value of the model solutions containing Pb(II) and Cu(II) ions was set to the desired values in the pH range of 2-10 by using buffer solutions. The results in which the optimum recovery was found as 5.5 is given in Figure 2. At high solution pH value, metal ions bind to OH− groups in the solution and cause precipitation. The pH value of 5.5 was applied for magnetic solid phase extraction of Pb(II) and Cu(II) on the magnetic M-MWCNTs in further studies.

### 3.2. Effect of the adsorbent amount

**Figure 3:**
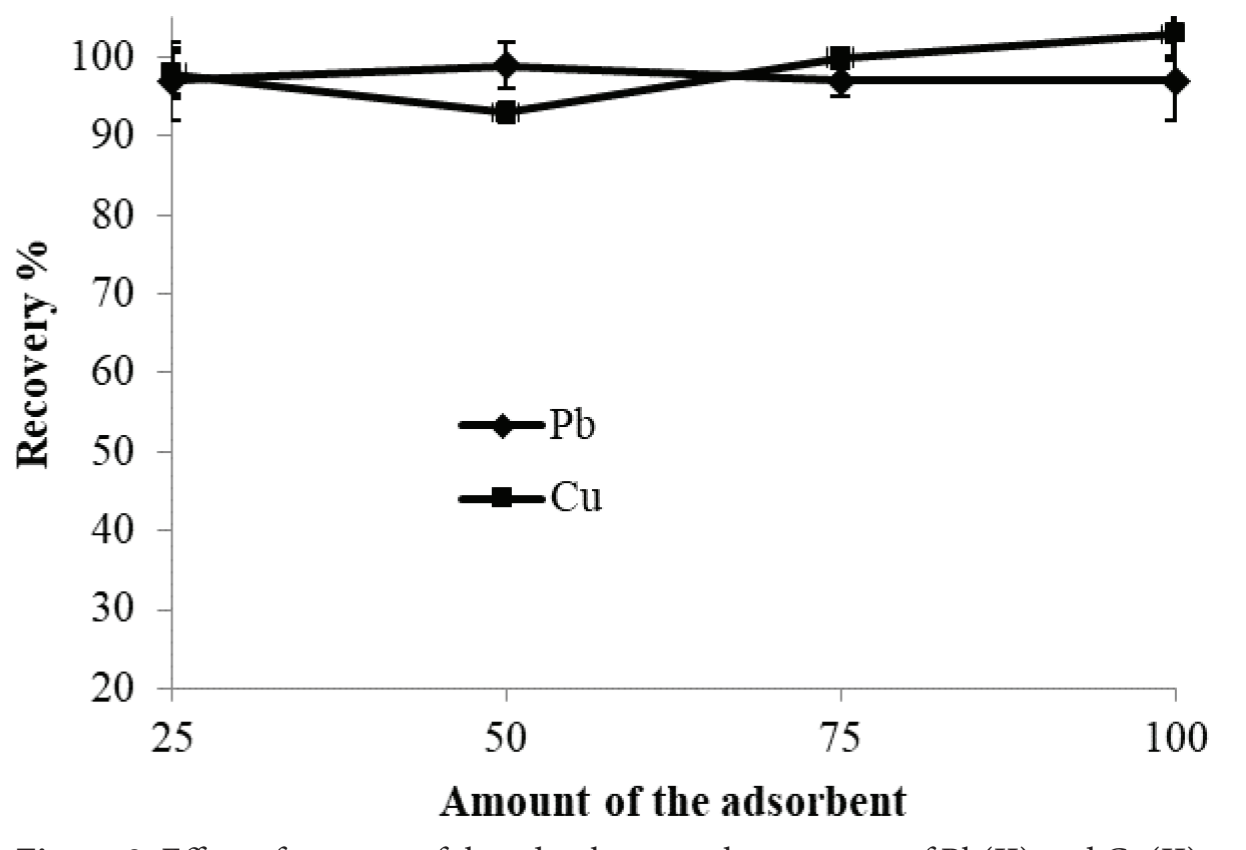
Effect of amount of the adsorbent on the recovery of Pb(II) and Cu(II).

The adsorbent amount in the SPE methods is a significant parameter for the adsorption of the analytes. Therefore, the effects of the quantities of the magnetic M-MWCNTs impregnated with PAN varied from 25 to 100 mg were investigated on the recovery values of the analyte ions. According to the obtained results given in Figure 3, it was determined that Pb(II) and Cu(II) were recovered quantitatively on adsorbent with adsorbent amount of 75-100 mg, and 75 mg of adsorbent was used for the following experiments.

### 3.3. Eluent type, concentration and volume effects

It is important to use an eluent, which effectively removes the adsorbed analytes in the adsorption studies. Thus, various organic and inorganic solvents at different concentrations have to be tested to choose the optimum eluent type. For this purpose, many desorbing solutions including 0,5-2 M HCl, 1-2 M HNO_3_ and 1-3 M HNO_3_ in 10% acetone were checked in the volume range of 1-5 mL. It is found that 3 mL 3 M HNO_3_ in 10% acetone is the most appropriate solution for the desorption of the analytes from the adsorbent.

### 3.4. Effect of sample volume

**Figure 4:**
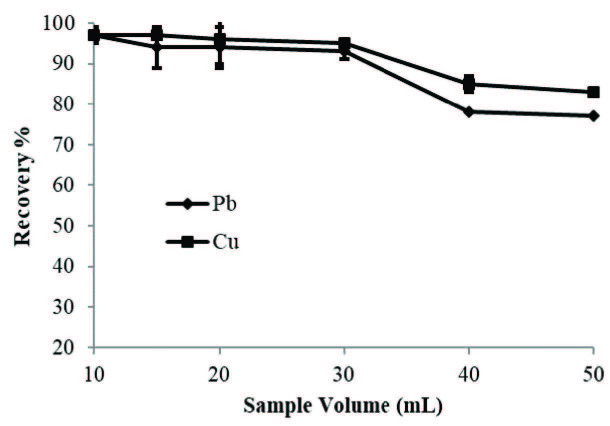
Effect of sample volume on the recovery of Pb(II) and Cu(II).

To investigate the sample breakthrough volume, a number of experiments were performed to optimize the sample volume in the range of 10-50 mL while keeping the other conditions as optimum. It can be concluded from the results shown in Figure 4 that the presented SPE method can be performed quantitatively up to 30 mL of a sample volume.

### 3.5. Effect of vortex time

To investigate the effect of vortex time on the efficiency of the adsorption process for the analyte ions, 0.5-2.0 min was worked, and the optimum vortex time was chosen as 1 min for further studies.

### 3.6. Matrix effect

The effects of some possible matrix ions in real samples, such as alkaline and earth alkaline metal ions, some anions on the recovery of Pb(II) and Cu(II) ions were examined by addition of various concentration of possible matrix ions to the model solutions keeping the other optimum conditions same as found before. The experimental results shown in Table 1 indicates that this magnetic solid phase extraction method can be used to determine Pb(II) and Cu(II) accurately in the different type of real samples without matrix effects.

**Table 1:** Effect of matrix ions on the recovery of Pb(II) and Cu(II), (n = 3).

Ion	Added as	Concentration (mg L^-1^)	Recovery, %	
			Pb	Cu
Na+	NaNO3	750	93±2	87±3
K+	KCl	750	91±2	93±2
Mg2+	Mg(NO3)2.6H2O	2500	95±2	103±1
Ca2+	Ca(NO3)2.4H2O	750	98±2	97±2
Fe^3+^	Fe(NO3)3.9H2O	1	91±2	95±2
Zn2+	Zn(NO3)2.6H2O	10	97±2	93±1
Mn2+	Mn(NO3)2.4H2O	1	88±4	90±4
Co2+	Co(NO3)2.6H2O	1	91±2	92±2
SO42-	Na2SO4	1250	98±3	102±1
Cl-	KCl	750	91±2	93±2

### 3.7. Analytical performance

Some analytical characteristics of the proposed method including preconcentration factor, limit of detection, and limit of quantification were listed in Table 2. The influence of sample volume on the solid phase extraction of analyte elements was investigated in the volume range of 10.0-50.0 mL. The extraction efficiencies of both of analytes were quantitative up to 30.0 mL of sample volume. The preconcentration factor was 10, when final volume is 3 mL. The inter-day and intra-day precision values of both analytes were below 4.0%.

**Table 2:** Some analytical characteristics of the method.

Parameters	Analytical feature of Pb	Analytical feature of Cu
Regression equation, C (µg L^-1^)	A = 0.008C-0.0009	A=0,037C+0,0008
Correlation coefficient (r)	0.999	0.999
LOD (3Sb/m) µg kg^-1^	16.6	18.9
LOQ µg kg^-1^	55.4	62.9
Preconcentration factor	10	10

The limit of detection (LOD = 3 × Sb/m Here, 3×Sb: Three times of the standard deviation of 10 runs measurements of blank solutions and m: the slope of the calibration curve) was found as 16.6 µg L^-1^ and 18.9 µg L^-1^, and the limit of quantification (LOQ = 10 × Sb/m Here, 10 x Sb: Ten times of the standard deviation of the blank solutions (n = 10), and m: the slope of the calibration curve) was found as 55.4 µg L^-1^ and 62.9 µg L^-1^ for Pb(II) and Cu(II), respectively.

### 3.8. Cu and Pb in real samples

The developed preconcentration method was applied to the determination of Pb(II) and Cu(II) in various herb samples, and the method is also applied to certificated SPS-WW2 wastewater and NCS-DC 73349 (bush branches and leaves) samples. The results are given in Table 3. The method was also applied successfully to determine Pb(II) and Cu(II) in herbs and spices such as daisy, mint, sage, daphne leaves,*Viburnum opulus*, rosemary, heather, green tea, and paprika. The results were shown in Table 4. According to the results, only the green tea sample contains Pb with a concentration of 1.6 µg g^-1^ while the others have concentrations below the detection limit. The maximum level for lead in fresh herbs and leaf vegetables is 0.1 (mg kg^-1^ wet weight) according to Commission Regulations No 1881/2006 and No 629/2008. On the other hand, according to WHO, maximum permissible limit of copper is 0.1 mg L^-1^, and the Cu concentrations in the samples were found in the range of 1.9-11.7 µg g^-1^. The highest value was found for green tea sample.

**Table 3:** Certified material analysis results (n = 3).

Analyte	SPS-WW2 waste water, μg L^-1^			NCS-DC73349 Bush Branches and Leaves, μg g^-1^		
	Certificated value	Found	Recovery %	Certificated value	Found	Recovery %
Pb	500±3	530±2a	106	47±2	46.6±2.3	99
Cu	2000±10	2210±1	110	6.6±0.08	6.8±0.7	103

a mean ± standard deviation

**Table 4:** Pb(II) and Cu(II) contents of real samples (n = 4).

Sample	Concentration (µg g^-1^)	
	Pb	Cu
Daisy	BDLa	10.5±3.6b
Mint	BDL	10.3±1.3
Viburnum opulus	BDL	4.7±0.3
Paprika	BDL	5.1±0.7
Sage	BDL	4.1±0.7
Daphne leaves	BDL	4.5±1.8
Rosemary	BDL	3.9±1.1
Heather	BDL	1.9±0.5
Green tea	1.6±0.0	11.7±1.7

a BDL: Below Detection Limitb mean ± standard deviation

### 3.9. Comparison to other extraction procedures in literature

The presented procedure was compared with some other preconcentration procedures in literature [29,31-38]. The comparison is given in Table 5. The presented procedure shows generally and comparatively low detection limit for analyte elements with some exceptions.

**Table 5:** Comparison of the M-MWCNTs-PAN method with other methods for determination of Pb and Cu in real samples with FAAS.

Extraction method	Sample	LOD, μg L−1	PF	Ref
Magnetic solid phase extraction	Water	Pb:1.76	15	[29]
Magnetic solid phase extraction	Water, food	Pb: 2.3Cu: 2.34	37-40	[31]
Cloud point extraction	Water, food	Pb: 3.42Cu: 0.67	2525	[32]
Ionic liquid dispersive magnetic micro extraction	Water, plant, hair	Pb: 0.57	160	[33]
Solid phase extraction	Water, cereal	Cu: 1.5	13	[34]
Solid phase extraction	Water, herb, fish	Pb:170	250	[35]
MWCNTs- D2EHPA	Water, wastewater	Cu:50	25	[36]
Coprecipitation	Vegetable	Cu:18Pb:35.9	50	[37]
Vortex-assisted micro solid phase extraction	Water	Pb:42Cu:22	35	[38]
Magnetic solid phase extraction	Herb	Pb:16.6Cu:18.9	1010	This work

## 4. Conclusion

This work demonstrated a new environmentally friendly, simple, rapid, and low-cost method for the separation and detection of trace amounts of Pb(II) and Cu(II) in various samples when compared with the other solid phase extraction methods. In this method, new M-MCNTs was successfully synthesized and used as a favorable absorbent for VA-MSPE prior to FAAS determinations. By using this, SPE method Pb and Cu contents of daisy, mint, sage, daphne leaves,*Viburnum opulus,*rosemary, heather, green tea, and paprika samples were determined without interfering effects. One of the other advantages of the method was the removal of the adsorbent easily from the solution by only using a magnet in a very short time. Due to its advantages, this method can be successfully applied to water, food, and environmental samples containing complex matrix.
